# Physical Activity to Counter Age-Related Cognitive Decline: Benefits of Aerobic, Resistance, and Combined Training—A Narrative Review

**DOI:** 10.1186/s40798-025-00857-2

**Published:** 2025-05-17

**Authors:** Wissem Dhahbi, Walid Briki, Andreas Heissel, Lutz Schega, Ismail Dergaa, Noomen Guelmami, Abdelfatteh EL Omri, Helmi Chaabene

**Affiliations:** 1 Research Unit “Sport Sciences, Health and Movement”, High Institute of Sports and Physical Education of Kef, University of Jendouba, Kef, Tunisia; 2 Qatar Police Academy, Police College, Training Department, Doha, Qatar; 3 Centre Hospitalier de Grasse, Grasse, France; 4 Social and Preventive Medicine, Department of Sports and Health Sciences, Intra Faculty Unit “Cognitive Sciences”, Faculty of Human Science and Faculty of Health Sciences Brandenburg, Research Area Services Research and e-Health, Potsdam, Brandenburg Germany; 5 Department of Sport Science, Chair for Health and Physical Activity, Otto-von-Guericke University Magdeburg, Magdeburg, Germany; 6 High Institute of Sport and Physical Education of Ksar Said, University of Manouba, Manouba 2010, Tunisia; 7 Research Unit Physical Activity, Sport, and Health, UR18JS01, National Observatory of Sport, Tunis, Tunisia; 8 Postgraduate School of Public Health, Department of Health Sciences (DISSAL), University of Genoa, Genoa, Italy; 9 Department of Human and Social Sciences, High Institute of Sport and Physical Education of Kef, University of Jendouba, 7100 Kef, Tunisia; 10 Surgical Research Section, Department of Surgery, Hamad Medical Corporation, 3050 Doha, Qatar; 11 Université de Jendouba, Institut Supérieur de Sport et de l’Education Physique du Kef, 7100 Le Kef, Tunisia

**Keywords:** Physical exercise, Training modality, Mental health, Older adults, Cognition, Executive function

## Abstract

**Background:**

With the increase in life expectancy, age-related cognitive decline has become a prevalent concern. Physical activity (PA) is increasingly being recognized as a vital non-pharmacological strategy to counteract this decline. This review aimed to (i) critically evaluate and synthesize the impact of different PA and exercise modalities (aerobic, resistance, and concurrent training) on cognitive health and overall well-being in older adults, (ii) discuss the influence of exercise intensity on cognitive functions, and (iii) elucidate the potential mechanisms through which PA and exercise may enhance or mitigate cognitive performance among older adults.

**Main Body:**

An exhaustive analysis of peer-reviewed studies pertaining to PA/exercise and cognitive health in older adults from January 1970 to February 2025 was conducted using PubMed, Scopus, Web of Science, PsycINFO, and MEDLINE. There is compelling evidence that aerobic and resistance training (RT) improve cognitive function and mental health in older adults, with benefits influenced by the type and intensity of exercise. Specifically, moderate-intensity aerobic exercise appears to bolster memory, executive functions, and mood regulation, potentially through increased cerebral blood flow, neurogenesis, and production of brain-derived neurotrophic factors in the hippocampus. Moderate-to-high-intensity RT acutely enhances visuospatial processing and executive functions, with chronic training promoting neurogenesis, possibly by stimulating insulin-like growth factor-1 and augmenting blood flow to the prefrontal cortex. Findings related to the effects of concurrent training on cognitive function and mental health are heterogeneous, with some studies reporting no significant impact and others revealing substantial improvements. However, emerging evidence indicates that the combination of concurrent training and cognitive tasks (i.e., dual tasks) is particularly effective, often outperforming aerobic exercise alone.

**Conclusions:**

Regular aerobic and RT performance is beneficial for older adults to mitigate cognitive decline and enhance their overall well-being. Specifically, engaging in moderate-intensity aerobic exercises and moderate-to-high-intensity RT is safe and effective in improving cognitive function and mental health in this demographic. These exercises, which can be conveniently incorporated into daily routines, effectively enhance mental agility, memory, executive function, and mood. The findings related to concurrent training are mixed, with emerging evidence indicating the effectiveness of combined concurrent and cognitive tasks on cognitive health and well-being in older adults.

**Key Points**

- Moderate-intensity aerobic exercise is associated with significant improvements in cognitive function, mood regulation, and overall well-being in older adults. These benefits are linked to structural and functional changes in the brain such as increased hippocampal volume and elevated levels of brain-derived neurotrophic factor.

- Moderate-to-high-intensity resistance training, both in acute and chronic forms, enhances cognitive performance in older adults, particularly in executive functions and visuospatial processing. Cognitive benefits, including improvements in information-processing speed, attention, and memory, can be sustained through regular training.

- The effects of concurrent resistance and aerobic training on cognitive function in older adults are mixed. However, combining concurrent training with cognitive tasks (i.e., dual-task training) is particularly effective and often outperforms aerobic exercise alone.

- Cognitive and well-being improvements from aerobic and resistance training are mediated by mechanisms such as increased cerebral blood flow and oxygen delivery, enhanced neurogenesis, reduced oxidative stress and inflammation, and positive hormonal changes.

- While the optimal exercise dosage for promoting cognitive health in older adults remains undetermined, empirical evidence indicates a positive correlation between increased exercise dosage and cognitive health improvements.

## Background

Age-related cognitive decline is a significant public health concern, characterized by decrements in key cognitive domains such as memory, executive function, and processing speed [1]. While a commonly cited figure suggests an approximate 5% decline in cognitive function per decade after the age of 40 [2], this generalization requires further investigation and refinement due to the complex interplay of factors influencing cognitive aging. There is a growing body of evidence that highlights the crucial role of physical activity as a modifiable lifestyle factor with the potential to mitigate age-related cognitive decline. Specifically, studies examining the differential effects of aerobic exercise, resistance training (RT), and concurrent training protocols have provided critical insights into tailored interventions aimed at optimizing cognitive function and mental well-being among older adults. Studies conducted from 2022 to 2025 have begun to elucidate distinct dose–response relationships between specific exercise parameters and cognitive outcomes. For instance, findings indicate that moderate-intensity aerobic exercise (50–70% of maximal heart rate—MHR) performed 3–7 times per week may yield optimal benefits for executive functions, while progressive RT (40–80% of one repetition maximum—1RM) performed twice weekly appears to be particularly beneficial for enhancing memory and attentional processes [3, 4]. As a result, individuals become more vulnerable to neurodegenerative conditions and various forms of dementia, including vascular dementia [5] and Alzheimer’s disease [6, 7]. Age-related declines in executive functions frequently manifest as reduced performance in tasks requiring attention-switching (e.g., multitasking) and greater difficulty in performing instrumental activities of daily living [8]. Moreover, ageing can result in other cognitive issues, such as longer response times, slower information processing speeds, and reduced inhibitory control [9]. These executive processes rely heavily on the frontal cortex, the volume and function of which decrease with age [9]. Additionally, language comprehension, which relies on working memory, tends to decline with ageing [9].

Implementation of systematically structured, evidence-based physical activity (PA) programs, including aerobic, resistance, and multicomponent training, has emerged as a critical strategy to counteract age-related declines in cognitive and physical function [10]. Regular engagement in PA has been shown to enhance cognitive performance in older adults, with concomitant benefits for overall brain health [11], and longitudinal studies demonstrating dose-dependent effects on cognitive health. Additionally, PA serves as a preventive measure against age-related cognitive decline [12]. While recent meta-analytic evidence suggests that long-term physical exercise interventions alone may have modest effects on cognitive function, multi-domain interventions incorporating physical exercise demonstrate more consistent benefits for global cognitive function in older adults [3] and appear to slow certain aspects of the cognitive ageing process [13]. Brain regions vulnerable to age-related atrophy exhibit notable structural and functional changes in response to PA, emphasizing its significant role in sustaining and enhancing brain functions [14, 15]. These neuroplastic changes are associated with improvements in cognitive performance, suggesting a direct link between PA and cognitive health in older adults [14]. Notably, epidemiological studies have consistently pointed towards a reduced risk of mild cognitive impairment and dementia in older adults who consistently engaged in regular PA throughout their lifetime [14, 16]. While the effects of PA on mental well-being are also significant, they often occur in parallel with cognitive benefits, highlighting the interconnected nature of cognitive and emotional health in the ageing brain [17, 18]. This integrated perspective on PA’s effects provides a more comprehensive understanding of its potential to maintain and improve cognitive function in older adults. While aerobic training has been extensively studied, recent evidence highlights the differential impacts of RT and concurrent training modalities on cognitive health [14, 19–22]. Specifically, RT has been shown to enhance attention and visuospatial processing [23], while concurrent training improves executive function and processing speed through synergistic mechanisms such as increased cerebral blood flow and neurotrophic factor release [24, 25]. This highlights the necessity for a detailed exploration of the specific modalities, intensities, and durations of each exercise type. However, the specific impact of exercise modality and intensity on cognitive health in older adults is less explored and requires a critical synthesis. There are indications that the intensity of various exercise modalities, such as aerobic, resistance, and combined training, can significantly influence cognitive health [26–31]. For instance, RT has been associated with potential cognitive benefits in older adults [32–36]. These studies emphasise the potential of RT to counteract age-related mitochondrial impairment in skeletal muscle and enhance cognitive function. In addition, the effects of combined aerobic and RT (i.e., concurrent training) on cognitive functions in older adults have been examined [37–41]. While studies indicate that concurrent training can positively influence cognitive functions in ageing populations, it is crucial to consider official guidelines on this matter. The World Health Organization (WHO) recommends regular PA for older adults to maintain cognitive health, emphasizing both aerobic exercise and strength training [42]. However, specific recommendations regarding exercise intensity across different training modalities for cognitive health preservation remain less defined. Despite the growing body of literature, there remains a notable lack of reviews that systematically compare the efficacy of different exercise modalities across distinct cognitive domains, such as executive function, memory, and attention [25, 43]. This gap underscores the need for a comprehensive synthesis of existing literature on the effects of various exercise intensities and modalities on cognitive function in older adults, which could inform future guidelines and interventions aimed at preserving cognitive vitality in ageing populations.

Currently, descriptive synthesis regarding exercise modalities and intensities in relation to cognitive function in older adults is lacking. This review aimed to provide a comprehensive analysis of the effects of various exercise modalities on cognitive health in older adults, addressing key gaps in the existing literature. Specifically, our objectives were to (i) critically synthesize the effects of aerobic, resistance, and concurrent training on cognitive function in older adults, (ii) evaluate the impact of exercise intensity on cognitive outcomes in this population, and (iii) elucidate the potential mechanisms through which PA may enhance or mitigate decline in cognitive performance among older adults. While our primary focus is on cognitively healthy older adults, we also include studies addressing those with pre-existing cognitive impairments, categorized as “mild” or “severe”, to provide a more nuanced understanding of exercise effects across varying cognitive states. By synthesizing findings from diverse exercise interventions and considering both cognitively healthy and impaired populations, this review seeks to provide actionable insights for researchers, clinicians, and policymakers seeking to develop evidence-based strategies for preserving and enhancing cognitive health in aging populations.

## Search Strategy

A comprehensive literature search was conducted in PubMed, Scopus, Web of Science, PsycINFO, and MEDLINE, covering publications from January 1970 to February 2025. Two researchers (W.D. and W.B.) conducted the search independently using Boolean operators to combine keywords. Duplicate records were removed before screening. The search strategy employed the following key terms: (exercise OR “physical training” OR “aerobic exercise” OR “exercise intensity” OR cycling OR swimming OR jogging OR Yoga OR running OR “strength exercise” OR “resistance exercise” OR “weight-lifting exercise” OR “weight-bearing exercise” OR “power training” OR walking OR “body-weight exercise” OR HIIT) AND (cognition OR “cognitive function” OR “brain ageing” OR neurodegeneration OR dementia OR “cognitive decline”) AND (“older adults” OR “older individuals” OR elderly OR ageing OR ageing). Study designs were limited to peer-reviewed randomized controlled trials (RCTs), high-quality cross-sectional studies with matched controls, and systematic reviews published in English. To ensure contemporary relevance, particular emphasis was placed on meta-analyses and RCTs published between 2020 and 2025, focusing on modality-specific effects, dose–response relationships, and individual variability in exercise response [10, 25, 44]. This includes specifying the intensity, duration, and frequency of each modality to elucidate their differential impacts on cognitive domains such as executive function, memory, and attention. The target population comprised adults aged ≥ 65 years, stratified by cognitive status (normal cognition, mild cognitive impairment, or dementia) according to standardized criteria [24].

## Aerobic Exercise and Cognitive Functions in Older Adults

Exercise prescription parameters for aerobic training should be carefully considered to optimize cognitive benefits. Recent evidence indicates that session duration (20–60 min), frequency (3–7 sessions/week), and intensity progression (starting at 40–50% maximal heart rate—MHR, and gradually increasing to 60–70%) are key factors determining intervention effectiveness [3]. Additionally, exercise mode selection should account for individual fitness levels and comorbidities, with walking, cycling, and water-based activities being particularly well-tolerated and accessible options for most older adults [4]. These parameters underscore the importance of personalized exercise prescriptions to maximize adherence and cognitive outcomes.

Aerobic exercise, a fundamental component of PA, is instrumental in preserving overall health and wellness [45]. Often termed “cardio”, aerobic exercise involves rhythmic and sustained engagement of large muscle groups [46]. It crucially depends on oxygen to fulfil the energy requirements during physical exertion. Aerobic exercises include activities such as walking, cycling, swimming, and jogging, which elevate the heart rate and augment oxygen consumption (e.g., 50–85% MHR), thereby fostering cardiovascular health [47].

Aerobic exercise not only enhances cognitive function but also positively influences mood regulation, which may indirectly support cognitive health. Research indicates that depressive symptoms can negatively impact cognitive performance in older adults [48], highlighting the interconnectedness of mental and cognitive health. Aerobic exercise has been shown to reduce depressive symptoms and improve mood, with some studies suggesting it is as effective as standard antidepressant medication in treating major depressive episodes [49, 50]. These mood improvements may indirectly enhance cognitive function by reducing stress, enhancing neuroplasticity, and promoting overall brain health [51].

Consistent research highlights the positive impact of aerobic exercise on cognitive abilities [52]. Notably, aerobic activities enhance insulin signalling in the brain, leading to improved glucose metabolism and overall brain health [53, 54]. Aerobic exercise also induces vasodilation, resulting in increased blood flow to cerebral regions, which may improve cognitive function by nourishing the brain tissue [55]. Long-term aerobic exercise is associated with significant structural changes in the brain, including increased volumes of the anterior hippocampus and medial temporal lobe, regions linked with memory and learning [56]. Furthermore, aerobic exercise appears to reduce extracellular amyloid-β accumulation, a characteristic feature of neurodegenerative diseases such as Alzheimer’s disease [26, 52].

### Low-Intensity Aerobic Exercise and Cognitive Functions in Older Adults

Low-intensity aerobic exercise (e.g., 50–60% MHR, 3–5 rate of perceived exertion [0–10 scale]—RPE, walking at 100 steps per minute or 1.6–3.0 metabolic equivalent of a task—METs) [27] programs have shown considerable promise in promoting cognitive health in healthy older adults (e.g., significant improvements in spatial memory and mood regulation) [57–59]. Moreover, recent studies have extended these findings to individuals with cognitive impairments. Specifically, low-intensity aerobic exercise programs have shown potential in mitigating progressive cognitive deficits observed in conditions such as mild-to-moderate Alzheimer’s disease [60, 61]. This suggests that these exercise regimens could be beneficial for cognitive health, both in healthy older individuals and those with cognitive disorders.

Varma et al. [58] investigated the relationship between daily walking activity and hippocampal volume in non-demented women aged over 60 years, revealing a strong positive association between low-intensity walking and hippocampal volume. Low-intensity walking was defined as activity at less than 100 steps per minute [58]. The same authors concluded that even modest increases in low-intensity walking were associated with measurable increases in hippocampal volume [58]. This relationship was consistent across various measures of walking activity, including daily step count, total walking time, and duration of walking bouts [58]. Similarly, Erickson et al. [62] performed a comparative analysis of cognitive and physiological impacts in sedentary, non-demented older adults engaging in either aerobic exercise (i.e., exercise regimen involving walking, starting at 10 min and increasing by 5-min increments weekly until reaching a 40-min duration by the seventh week; the target heart rate zone was set at 50–60% of the maximum heart rate reserve) or mobility exercises (two muscle-toning exercises and two balance exercises, one yoga sequence, and one exercise of the participants’ choice) [62]. The study results indicated significant improvements in cognitive functions, such as spatial memory, increased anterior hippocampal volume, and elevated levels of brain-derived neurotrophic factor (BDNF) in the hippocampus. Importantly, these beneficial effects were observed exclusively in the aerobic exercise group, further substantiated by an increase in maximal oxygen uptake (VO_2max_) [62]. This suggests the potential superiority of aerobic exercise over stretching exercise in enhancing cognitive and physiological functions in older adults.

Despite the accumulation of promising insights, there remains a divergence of opinions regarding the impact of low-intensity aerobic exercise on cognitive functions. Ferreira et al. [63] demonstrated that a low-intensity aerobic training program led to improvements in cognitive functions, specifically in abstraction and mental flexibility. Lautenschlager and Cox [64] suggested a volume-dependent effect on cognitive performance. This finding suggests the existence of a volume threshold that exercise programs must exceed to elicit significant cognitive benefits. This aspect requires further exploration.

**Fig. 1:**
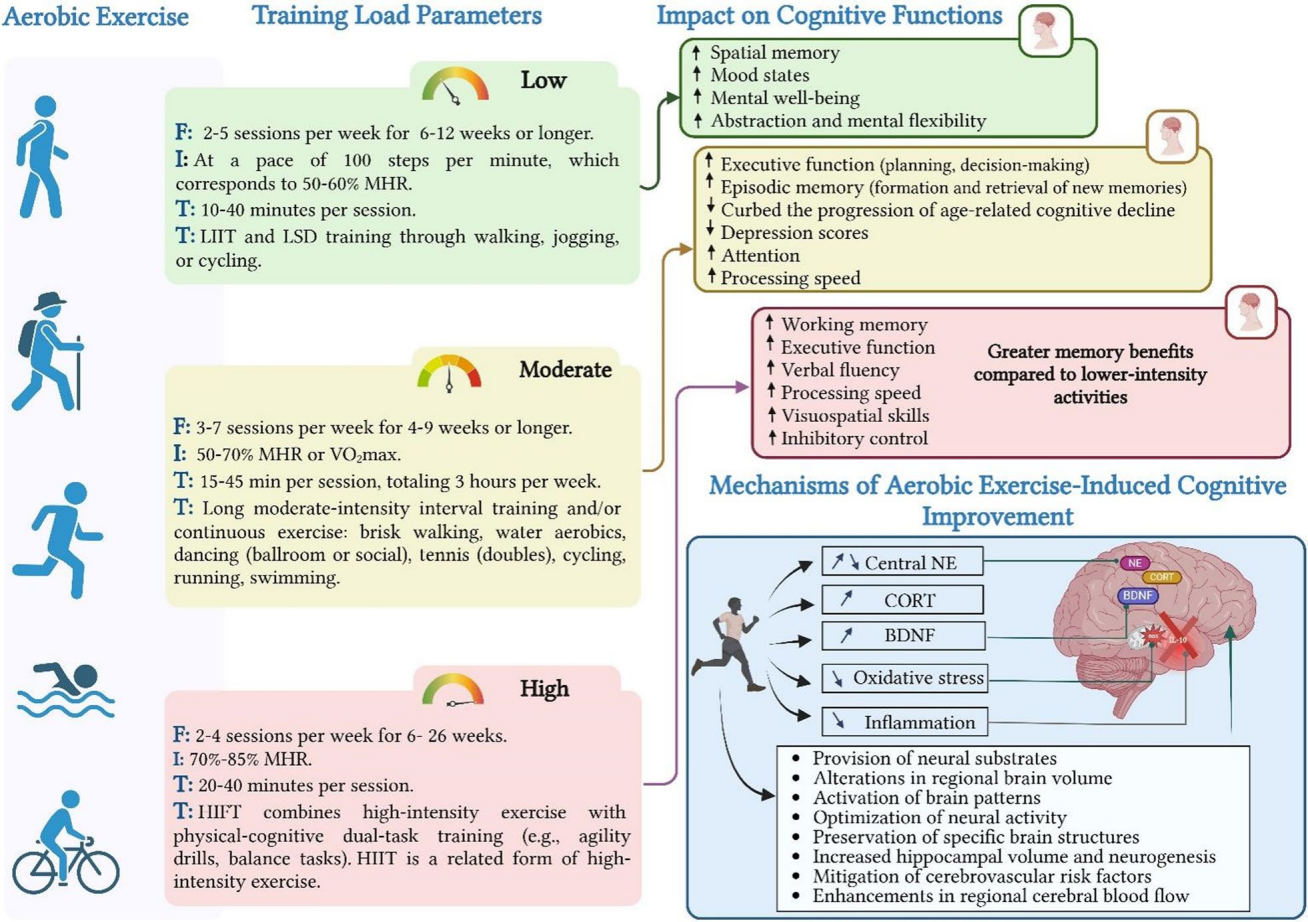
The impact of different aerobic exercise intensities on older adults’ cognitive health. *MHR* maximum heart rate, *HIIT* high-intensity interval training, *HIFT* high-intensity functional training, *LIIT* low-intensity interval training, *LSD training* long, slow distance training, *FITT* frequency, intensity, time, type, *BDNF* brain-derived neurotrophic factor, *CORT* Cortisol, *NE* Norepinephrine, *VO*_*2max*_ maximal oxygen consumption

In summary, low-intensity aerobic exercise has shown significant potential in enhancing cognitive function and overall well-being in older adults, regardless of their cognitive health status [65, 66]. These exercises have been associated with improvements in cognitive function [64], increased hippocampal volume, and elevated hippocampal BDNF levels [59]. Moreover, they have demonstrated a positive impact on mood regulation, particularly in individuals with depressive disorders [59] (Fig. 1).

### Moderate-Intensity Aerobic Exercise and Cognitive Functions in Older Adults

Extensive research has investigated the impact of moderate-intensity aerobic exercise on cognitive function. Modalities such as running, cycling, and swimming, performed at 60–70% of maximum heart rate reserve (corresponding to a rating of 5–6 on the 0–10 Rate of Perceived Exertion scale—RPE or 3.0–6.0 METs), have been shown to enhance executive function, memory, and mood regulation. These benefits are potentially mediated by mechanisms including increased hippocampal volume, elevated BDNF levels, and improved cerebral perfusion [21, 65]. Recent evidence (2022–2025) has begun to delineate distinct dose–response relationships between specific exercise parameters and cognitive outcomes [15, 18, 19, 25, 63, 67–73]. Earlier research broadly supports the benefits of moderate-intensity aerobic exercise (50–70% of maximal heart rate—MHR) performed 3–7 times per week for executive function. Therefore, further research is needed to refine these recommendations and elucidate the optimal exercise prescriptions for various cognitive domains. Cross-sectional studies have provided initial insights into this relationship. For instance, Bixby et al. [74] found a positive correlation between moderate-intensity aerobic exercise and executive function in older adults. However, cross-sectional studies offer less trustworthy evidence, owing to their observational nature. Intervention studies, however, provide stronger evidence of causality. Regular engagement in moderate-intensity aerobic activities, for instance, 30 min on most days of the week, has been associated with enhancements in executive function and memory in healthy older adults [75, 76].

Muscari et al. [77] demonstrated that a 12-month moderate-intensity aerobic training program (equivalent to three hours per week) effectively slowed the progression of age-related cognitive decline in a cohort of older adults. Similarly, Rhodes et al. [78] demonstrated cognitive improvements in executive function and memory in older adults who participated in 15-min moderate-intensity aerobic exercise sessions three times a week for 12 weeks. This intervention also resulted in reduced depressive symptoms, highlighting the broader benefits of moderate-intensity aerobic exercise [78]. Such moderate aerobic activities have also been found to improve cognition in older adults with mild cognitive impairment [79]. Likewise, routine interventions involving moderate-intensity aerobic exercise have shown potential as an effective strategy to slow the progression of mild cognitive impairment [80]. These cognitive enhancements are associated with observable structural and functional changes in the brain, including reduced brain atrophy rates and increased hippocampal efficacy (e.g., improvement in forming new memories, spatial learning and spatial memory, and neural connections within the hippocampus and between it and other brain regions) [81, 82].

In summary, moderate-intensity aerobic exercise has been extensively studied for its impact on cognitive function in older adults. Regular aerobic exercise enhances executive function, memory, and mood regulation while mitigating age-related cognitive decline. These benefits are mediated by mechanisms such as increased hippocampal volume, neurogenesis, and elevated BDNF levels. These benefits are observed in both healthy older adults and those with mild cognitive impairment, and are associated with observable structural and functional changes in the brain. HIIT has been shown to significantly improve working memory and verbal fluency, likely mediated by increased cerebral blood flow and neurotrophic factor release. Therefore, the incorporation of moderate-intensity aerobic exercise programs into strategies to improve cognitive health and well-being is highly recommended (Fig. 1).

### High-Intensity Aerobic Exercise and Cognitive Functions in Older Adults

The literature related to the impact of high-intensity aerobic exercise (i.e., 75–90% of maximum heart rate reserve, >6.0 METs) on cognitive function in older adults is heterogeneous. Recent evidence indicates that while moderate-intensity exercise produces beneficial adaptations, higher-intensity exercise protocols may induce greater and distinct adaptations in brain structure and function. In this sense, existing evidence indicates that HIIT and high-intensity functional training (HIFT) result in benefits for working memory, executive function, and verbal fluency in healthy older adults [55, 72], with potentially superior outcomes compared to moderate-intensity protocols in terms of BDNF upregulation and cerebral blood flow [71, 72]. These intensity-dependent adaptations appear to follow a dose–response relationship, where higher intensities may optimize cognitive benefits through distinct physiological mechanisms [25]. Notably, cardiorespiratory fitness seems to play a crucial role, with higher fitness levels associated with better cognitive function even in the absence of overall cognitive improvement [73, 83]. Interestingly, combining high-intensity exercise with motor-cognitive dual-task training has been shown to enhance processing speed, visuospatial skills, and inhibitory control, likely mediated by increased cerebral blood flow and the release of neurotrophic factors [10, 72, 84]. This suggests that a combined approach targeting both physical and cognitive challenges may be particularly beneficial. However, individual variations in response to exercise interventions, the effectiveness of different exercise modalities, and the choice of cognitive assessments all require further investigation [25, 70].

While some studies recommend moderate-intensity exercise as a potentially safer and more universally effective approach for cognitive enhancement in older adults [71], HIIT appears promising for enhancing cognitive flexibility compared with other training methods [85]. The enhanced cognitive benefits observed with higher exercise intensities may be attributed to greater increases in neuroplasticity markers, improved cerebral perfusion, and stronger cardiovascular adaptations [85]. However, individual fitness levels and health status should be considered when prescribing exercise intensity, with a gradual progression from moderate to higher intensities recommended for older adults [10]. For example, a study by Baker et al. [86] demonstrated significant improvements in executive function among healthy older adults who participated in a 6-month running program at high intensity (85% MHR) for four days per week. Similar benefits have been observed in individuals with amnestic mild cognitive impairment [79]. Durante and Ainsworth [87] found that higher-intensity activities offered greater memory benefits than lower-intensity activities in older adults.

In summary, high-intensity aerobic exercise demonstrates variable effects on cognitive function in older adults, with some studies reporting significant improvements in specific cognitive domains while others show no overall cognitive enhancements. Some studies have reported cognitive benefits in specific domains, such as working memory, executive function, and verbal fluency, following HIIT or HIFT. However, other studies have found no significant overall cognitive improvements. High-intensity aerobic exercise, particularly HIIT, has been shown to enhance working memory and verbal fluency, likely mediated by increased cerebral blood flow and neurotrophic factor release [10, 71, 85]. Combining high-intensity aerobic exercise with physical-cognitive dual-task training may enhance processing speed, visuospatial skills, and inhibitory control. Despite these findings, individual responses to exercise interventions, effectiveness of different exercise modalities, and choice of cognitive assessments require further investigation. Although moderate-intensity exercise is often recommended as a safer and more universally effective approach for cognitive enhancement, high-intensity activities have shown promise in enhancing cognitive flexibility and memory. However, the lack of consistent evidence makes definitive conclusions premature (Fig. 1).

## Resistance Exercise and Cognitive Functions in Older Adults

Research investigating the effects of RT on cognitive functions in older adults is expanding, with emerging evidence indicating promising results. RT, a specialized form of physical conditioning that includes a range of modalities such as weight machines, free weights, elastic bands, medicine balls, and plyometrics [88], has been demonstrated to potentially enhance cognitive functions [14]. RT prescription should follow evidence-based guidelines: 2–3 sessions per week, 8–10 exercises targeting major muscle groups, 2–3 sets of 8–12 repetitions at 40–80% RM, with adequate rest intervals (1–2 min between sets). Progressive overload should be implemented by gradually increasing the intensity (5–10% when participants can complete current workload with proper form) rather than repetitions [3, 4]. Exercise selection should prioritize compound movements that challenge both muscle strength and balance [24]. Specifically, studies by Chang et al. [89] and Colzato et al. [90] highlighted the potential of both acute and chronic resistance exercises to improve cognitive performance. However, these studies did not provide specific details or accurately quantify the intensity of RT exercises used, making it challenging to determine the most effective modalities. In contrast, the dose–response relationship between the intensity of RT and cognitive function in older individuals has become a topic of interest in recent years.

### Acute Effects of Resistance Exercise on Cognitive Functions in Older Adults

Prior research suggests that RT can acutely enhance or maintain cognitive performance in older adults, particularly in visuospatial processing [68]. Studies reporting non-significant results underscore the critical role of training parameters, particularly intensity (e.g., 70–80% 1RM), duration (≥6 months), and frequency (2–3 sessions/week), in eliciting cognitive benefits [91, 92]. The acute effects of RT on cognitive performance are primarily mediated by increased cerebral blood flow and enhanced neurotrophic factor release [23]. Specifically, RT performed at higher intensities (70–80% 1RM) induces greater cognitive benefits compared to lower intensities, likely due to increased insulin-like growth factor-1 (IGF-1) and cerebral blood flow [92]. Therefore, to optimize the acute cognitive benefits of RT training in older adults, a balanced approach that considers all three training parameters is crucial [93]. Recent studies [23, 94, 95] have substantiated that RT generates acute improvements in executive functions, including attention, working memory, problem-solving capabilities, cognitive flexibility, and verbal fluency. A study by Chang et al. [23] involving older adults subjected to acute resistance exercise (7 exercises, 2 sets, 70% 1RM, 10 repetitions) revealed a significant improvement in the performance of the exercise group in all Stroop tests compared to the control group. The study further demonstrated that RT, particularly when performed at moderate-to-high intensities, not only enhances general cognition but also induces significant improvements in executive control, working memory, and visuospatial processing [23]. Hsieh et al. [94] conducted observations across different age groups and found that RT (70% 1RM) positively impacted the working memory of both young males (21–30 years old) and older adults (65–72 years old), with a more pronounced effect on the latter for tasks requiring higher memory. Recent findings suggest that RT performed at higher intensities (70–80% 1RM) induces greater cognitive benefits compared to lower intensities, likely due to increased IGF-1 and cerebral blood flow [92]. Furthermore, Hsieh et al. [95] examined the variance in attention between males (21–30 years old) and older adults (65–69 years old) post-RT and reported an improvement in attention, indicating that the acute cognitive benefits of RT do not diminish with age.

Chang et al. [23] revealed that cognitive tasks performed within 5 min post-RT tend to have a detrimental impact, likely due to exercise-induced tachycardia and hyperthermia. However, cognitive tasks administered 11–20 min post-RT exhibited beneficial effects (Cohen’s d ≈ 0.2), suggesting that acute cognitive benefits of RT manifest approximately 10 min after exercise cessation and gradually diminish thereafter [96]. Factors such as exercise intensity, volume, duration, timing of cognitive testing, and participants’ health status significantly influence cognitive performance [91]. Supporting this, Sudo et al. [97] noted enhanced attention and information processing speed when cognitive functions were assessed at 10 and 30 min post-exercise, with pronounced benefits at the 10-min mark. Chow et al. [98] reported significant improvements in visuospatial processing and working memory 10 min post-RT. Additionally, Vonk et al. [99] found marked improvements in reaction time and executive function at 10 and 20 min post-exercise, with optimal benefits observed within this timeframe.

**Fig. 2:**
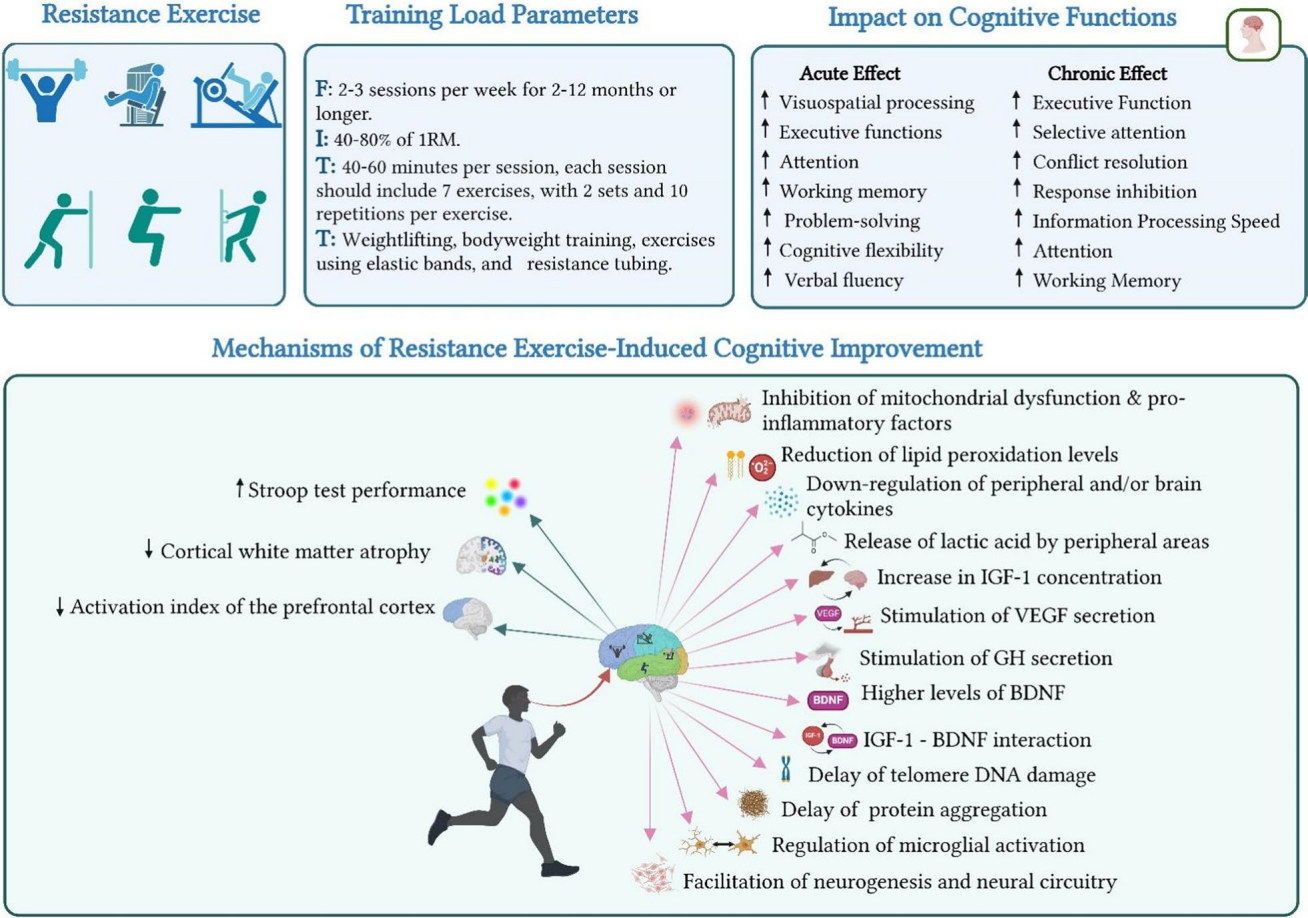
The acute and chronic impact of resistance exercise on older adults’ cognitive health. *FITT* frequency, intensity, time, type, *1RM* one repetition maximum, *IGF-1* insulin-like growth factor 1, *BDNF* brain-derived neurotrophic factor, *VEGF* vascular endothelial growth factor, *GH* growth hormone, *DNA* Deoxyribonucleic acid

In summary, RT has been shown to acutely enhance visuospatial processing and executive functions, with underlying mechanisms including increased cerebral blood flow, neurotrophic factor release, and reduced inflammation. However, the impact of RT depends on training parameters, such as intensity, duration, and frequency. While RT can be immediately beneficial for cognitive performance, the optimal window appears to be 10–20 min after exercise cessation. Therefore, a balanced approach to RT and careful timing of cognitive testing are essential to optimize the acute cognitive benefits of RT in healthy older adults (Fig. 2).

### Chronic Effects of Resistance Training on Cognitive Functions in Older Adults

Long-term RT induces cognitive enhancement through multiple interacting pathways, with chronic adaptations exceeding acute responses [100]. More specifically, experiments by Coelho et al. [100] demonstrated a 65.2% increase in plasma BDNF in elderly women following a 10-week lower-limb RT program performed thrice weekly. This aligns with other studies [14, 26, 101] that proposed that RT induces peripheral BDNF elevation, which in turn contributes to enhancing resilience against age-related brain damage and neurodegeneration. Furthermore, Chang et al. [102] suggested that to significantly enhance cognitive function in older adults (including information processing speed and attention) and to sustain these benefits over time, RT should be performed at least twice weekly for a duration of 2–12 months. Nagamatsu et al. [103] revealed that biweekly RT for six months (with intensity increasing from 40 to 80% 1RM) could improve selective attention, conflict resolution, and associative memory in older adults. Liu-Ambrose et al. [104] also reported that moderate-to high-intensity RT (70–80% 1RM, 6–8 repetitions) performed once or twice weekly for 12 months could enhance these cognitive functions. Finally, Tsai et al. [92] conducted a study where they randomly assigned 48 healthy older men to either an RT group (75–80% 1RM, 10 repetitions) or a control group. The exercise regimen was administered three times weekly for 12 months. The findings revealed a significant decline in the accuracy rate and P3a/P3b amplitudes within the oddball task in the control group after 12 months. Conversely, the exercise group demonstrated improved reaction times and sustained P3a/P3b amplitudes. Additionally, the exercise group exhibited increased insulin-like-growth factor-1 (IGF-1) and decreased homocysteine levels. These findings suggest that regular RT may be a promising strategy to mitigate cognitive decline in healthy older adults, which is potentially mediated by IGF-1.

Several studies have examined the effect of RT on cognitive functions in older adults. Singh et al. [105] found that a six-month high-intensity progressive RT regimen, performed two-to-three times weekly by older adults (70 years) with mild cognitive impairment, significantly improved global cognitive functions. Similarly, Fallah et al. [106] conducted a study on independently living older women (65–75 years) and compared the effects of a 60-min RT regimen, which included 6–8 repetitions of two sets of isokinetic-based exercises and additional strength exercises, with balance and tone training. The RT group showed enhanced performance in the Stroop test, indicating improved executive function. In a separate study, Liu-Ambrose et al. [104] demonstrated that older women participating in a 12-month RT program performed one to two times weekly improved in various executive function assessments, including selective attention, conflict resolution [107], and MRI-measured brain regions related to response inhibition [14]. This was in contrast to their counterparts, who only engaged in balance and toning exercises. Furthermore, research has indicated that RT combined with cognitive tasks can lead to improvements in both physical and cognitive performance in older adults [19]. Additionally, moderate-to-high intensity RT, lasting for at least 6 months, has been highlighted as more effective in enhancing cognitive function in older adults [67]. Evidence also suggests that RT programs with shorter session durations and higher frequencies may generate better cognitive outcomes in older adults with cognitive impairments [75].

These studies collectively accentuate the role of RT in fostering functional plasticity within brain regions intricately tied to executive function, particularly response inhibition [14]. The comprehensive perspective on the impact of RT on brain health extends further to the findings of Szalewska et al. [26]. Their study provided evidence suggesting that RT contributes to cerebral perfusion maintenance as well as the elevation of critical factors, such as BDNF, IGF-1, and vascular endothelial growth factor, all of which are associated with neurogenesis and brain insulin signalling enhancement.

Overall, the RT program has been shown to have pronounced effects on neurogenesis, particularly through the elevation of plasma BDNF levels, thereby enhancing resilience against age-related brain damage and neurodegeneration. The cognitive benefits of RT, including improvements in information-processing speed, attention, selective attention, conflict resolution, and associative memory, are significant and can be sustained over time with regular training. These benefits are particularly evident when RT is performed at moderate-to-high intensity, with a frequency of at least twice weekly for a duration of 2–12 months. The effectiveness of RT in enhancing cognitive function was also influenced by the duration and frequency of training sessions. RT may help mitigate cognitive decline in healthy older adults, potentially through IGF-1. It fosters plasticity in brain regions related to executive function, maintains cerebral perfusion, and enhances factors linked to neurogenesis and insulin signalling in the brain (Fig. 2). A key finding of this review is the differential impact of exercise modalities on specific cognitive domains. For instance, aerobic exercise has been shown to predominantly enhance memory and executive function, while resistance training demonstrates greater efficacy in improving attention and visuospatial processing [23, 37].

## Effects of Concurrent Strength and Aerobic Exercises on Cognitive Functions in Older Adults

Concurrent training represents an advanced intervention strategy that systematically integrates aerobic and resistance exercise modalities to enhance cognitive function in older adults [14]. This approach leverages synergistic adaptations through multiple physiological pathways, offering potential advantages over single-modality training programs. These combined interventions may have a more pronounced impact on cognitive functions than aerobic exercise alone [14, 43]. Evidence suggests that concurrent training can enhance cognition, particularly in older adults with mild cognitive impairment, with lasting effects observed at follow-up [69]. The combination of cognitive tasks with concurrent training (i.e., dual tasks) has been found to significantly enhance cognitive function in healthy older adults, indicating that this mixed training approach can improve both physical and cognitive performance [19, 22, 25, 84].

Lautenschlager et al. [30] highlighted the benefits of a 24-week home-based concurrent training (e.g., 24 weeks with three weekly sessions, each lasting 50 min), which led to improved performance on the Alzheimer Disease Assessment Scale-Cognitive Subscale among seniors with probable mild cognitive impairment. Building on these findings, Bossers et al. [108] investigated the effects of concurrent training versus aerobic training only on cognitive and physical-cognitive dual-task performance in older patients with dementia (mean age 86 years). They implemented a 9-week intervention that included two strength training sessions and two walking sessions per week, each lasting 30 min, at moderate-to-high intensity (RT; 12–30 repetitions body-weight exercises, at an RPE [12–15] < 12, with an attached weight of 0.5–1.5 kg; aerobic training: targeting a HR range of 50–85% of maximum). Bossers et al. [108] revealed that, compared to aerobic-only training, concurrent training was more effective in reducing cognitive and motor-cognitive dual-task decline in older patients with dementia.

Of note, not all of the available studies have revealed the positive effects of concurrent training on cognitive functions in older adults. For example, Makizako et al. [109] found no significant impact of concurrent training on attention-based dual-task performance in older adults with mild amnestic cognitive impairment. Conversely, Castillo Quezada et al. [110] reported that a 12-week concurrent training regimen (consisting of three 60-min sessions per week on alternate days) significantly enhanced cognitive function in healthy older adults, as indicated by increased Mini-Mental State Examination (MMSE) scores and IGF-1 levels. In the realm of cognitive training, dual-task training (DTT), which involves the simultaneous performance of two tasks, has proven effective in boosting cognitive function in the elderly population. Notably, DTT has been shown to be superior to aerobic exercise training alone [25], suggesting that a combination of concurrent training and cognitive tasks could be a potent strategy for promoting cognitive health in healthy older adults [111]. A meta-analysis by Colcombe and Kramer [37] indicated that concurrent training programs offer greater cognitive benefits to healthy, non-sedentary older adults than programs that only include aerobic training. However, these benefits are not unique to concurrent training. Another meta-analysis [25] and a study comparing traditional RT with RT combined with cognitive tasks [19] reported significant cognitive improvements in older adults following both types of exercise, underscoring the cognitive advantages of combining exercise with cognitive challenges. Finally, a systematic review by Su-Ju [111] found that concurrent training significantly improved cognitive function and physical fitness factors in elderly women with mild cognitive impairment after eight weeks.

**Fig. 3:**
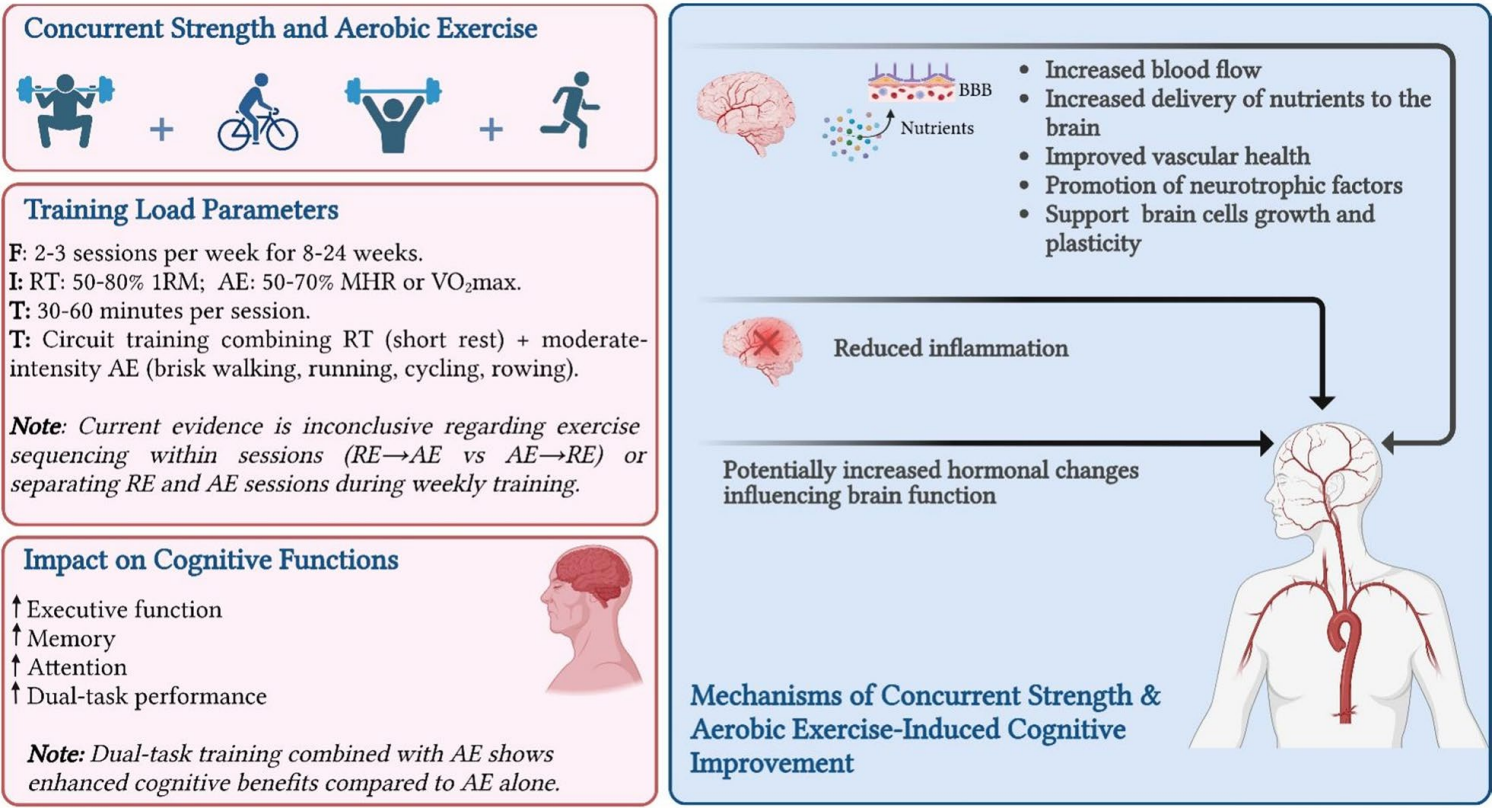
The impact of concurrent strength and aerobic exercise on older adults’ cognitive health. *RE* resistance exercise, *AE* aerobic exercise, *1RM* one repetition maximum; *VO*_*2max*_ maximal oxygen uptake, *MHR* maximum heart rate, *FITT* frequency, intensity, time, type, *BBB* blood brain barrier

Overall, the effects of concurrent resistance and aerobic training on cognitive function in older adults were multifaceted. While some studies have found no significant impact, others have reported substantial cognitive improvements. Dual-task training, when integrated with high-intensity concurrent exercise (e.g., 75–90% MHR for aerobic components and 70–80% 1RM for resistance components), demonstrates superior efficacy in enhancing executive function and processing speed. These benefits are mediated by synergistic effects on neuroplasticity, cerebral perfusion, and neurotrophic factor release [24, 25, 84]. The benefits extend to various populations, including healthy older adults, those with mild cognitive impairment, and those with dementia. The cognitive benefits of concurrent training are likely mediated by a combination of aerobic and resistance exercise mechanisms, including increased cerebral blood flow, neurotrophic factor release, and improved vascular health. The cognitive advantages of integrating exercise with cognitive challenges are evident across different types of exercise and not just concurrent training **(**Fig. 3**)**.

## The Potential Mechanisms of Physical Activity-Related, Cognitive Function Improvements in Older Adults

### Mechanisms of Aerobic Exercise-Induced Cognitive Improvement in Older Adults

Variations in heart rate, blood pressure, and respiratory patterns induced by aerobic-based PA have been shown to lead to fluctuations in central norepinephrine release [26, 112–114]. This cascade of physiological responses initiates a complex series of neurobiological adaptations that ultimately support cognitive enhancement. Effective modulation of cognitive functions through exercise may depend on optimal stimulation of locus coeruleus function, a pivotal regulator of arousal, decision-making, and attention [115, 116] that interfaces with successful cognitive function modulation through exercise [26, 117]. The release of cortisol, elicited by exercise-induced stress, affects learning and memory by influencing mineralocorticoid and glucocorticoid receptors [118, 119], with stimulation of mineralocorticoid receptors demonstrating efficacy in improving cognitive and affective functions [26].

Additional hypotheses bolstering the advantageous effects of aerobic exercise on cognitive function propose that the anterior corona radiata augments the provision of neural substrates to the brain, including neurotrophins, along with an enhanced supply of blood and oxygen [21, 65, 86]. Concurrently, these hypotheses propose a dual role of diminishing oxidative stress and inflammation, thereby safeguarding the hippocampal volume and promoting heightened neuronal plasticity. Furthermore, research has unequivocally demonstrated that aerobic exercise could enhance cognitive performance, coinciding with discernible alterations in regional brain volume [62, 120] and patterns of brain activation [121]. A systematic review with meta-analysis by Sáez de Asteasu et al. [122] found that in-hospital physical exercise and early rehabilitation programs effectively enhance cognitive function after discharge in older patients hospitalized for acute medical conditions. However, the benefits for preventing delirium were less clear, highlighting the need for targeted interventions. Notably, a study conducted by Marco et al. [123] revealed that aerobic exercise can contribute to the reduction of depression and anxiety symptoms in older adults. Insights gathered from neuroimaging studies have shown that heightened aerobic fitness is closely linked to optimization of neural activity within the prefrontal cortex and parietal regions associated with executive function [124, 125]. Furthermore, investigations have highlighted the preservation of specific brain structures, notably the right inferior frontal gyrus and precentral gyrus [126], in tandem with enhanced aortic elasticity [127].

Aerobic exercise, particularly of moderate intensity, has been linked to enhanced cognitive functions, primarily through its effects on the hippocampus, a brain region integral to memory consolidation [75, 76]. Regular aerobic exercise not only increases hippocampal volume but also promotes neurogenesis and the creation of new brain cells [76]. This leads to a reduction in age-related hippocampal atrophy, improved perfusion in brain regions associated with cognitive functions, and overall cognitive enhancement [51, 75, 76]. Cooper [128] proposed that the cognitive benefits of moderate-intensity aerobic exercise can be attributed to a moderate increase in peripheral norepinephrine concentration. However, it is important to note that an excessive surge in norepinephrine levels during intense exercise may have counterproductive effects on cognitive function. The cognitive benefits of moderate-intensity aerobic exercise have also been linked to its role in reducing cerebrovascular risk factors. Empirical evidence shows that a 12-week regimen of 60-min aerobic exercise sessions, conducted three times weekly at an intensity of 50–75% of the maximum heart rate, led to significant improvements in regional cerebral blood flow [20]. Specifically, this intervention generated improvements in the anterior cingulate region among healthy adults aged over 75 years [20]. Similarly, within a cohort of healthy adults aged 56–75 years, an identical training regimen led to increased cerebral blood flow in the hippocampal region, an area that is particularly susceptible to the effects of ageing and dementia [20].

In summary, moderate-intensity exercise seems to be particularly beneficial, potentially by optimizing activity in brain regions that are crucial for memory, decision-making, and executive function (Fig. 1).

### Mechanisms of Resistance Training-Induced Cognitive Improvement in Older Adults

Following RT intervention, peripheral or brain cytokines can be down-regulated [129]. This can delay telomere deoxyribonucleic acid (DNA) damage and protein aggregation, inhibit mitochondrial dysfunction and the expression of pro-inflammatory factors in ageing cells, reduce lipid peroxidation levels, and regulate microglial activation [129]. Consequently, this can prevent oxidative and inflammatory mechanisms from contributing to ageing, thereby delaying or preventing the onset of mild cognitive impairment and Alzheimer’s disease.

Indeed, as age advances, there is a significant decline in cognitive flexibility, which underpins complex decision-making processes [130]. Nevertheless, RT serves as a potent intervention, facilitating neurogenesis and neural circuitry through the provision of essential nutrients and energy [130]. While acute exercise can induce changes in cognitive ability, influenced by circulatory processes, such as blood flow and hormone levels, the long-term response may be attributed to structural adaptations. Studies have shown that following a 52-week period of RT, there is an increase in the concentration of IGF-1 in the peripheral serum of older individuals [131]. Another study [132] observed that after completing up to 52 weeks of RT, older individuals exhibited an increase in Stroop test performance, indicative of improved cognitive function, and a decrease in cortical white matter atrophy, suggesting reduced brain ageing. However, these changes were not observed in older individuals who performed non-aerobic exercises such as balance and conditioning exercises. Moreover, the authors of the same study [132] noted that brain activation decreased under relatively easy task conditions but increased under more challenging task conditions. Concurrently, enhancements have been observed in cognitive behavior, such as accuracy and reaction time in executive function tests, and cognitive function, such as P3 amplitude [131]. Coesee [133] showed that during the Stroop test, after a 16-week RT intervention in healthy older individuals, there was a decrease in the activation index of the prefrontal cortex, a reduction in oxyhemoglobin and total hemoglobin in the left prefrontal cortex (both compared with pre-test levels), and an improvement in cognitive task performance, specifically in reaction time.

Individuals who regularly engage in RT tend to have higher BDNF levels than those who are less active [101]. This can be attributed to the release of lactic acid from peripheral areas such as muscle tissue post-exercise, which serves as a “fuel” for cognitive processes [134]. This lactic acid, assisted by monocarboxylic acid transporters, crosses the blood–brain barrier [135, 136], instigating the activation of BDNF in the hippocampus and its release into the serum [137, 138]. This plays a crucial role in memory and learning, stimulating neural circuit function, and enhancing it. Additionally, BDNF produced following RT interacts directly or indirectly with IGF-1 [135, 136, 139]. This interaction aids the brain in increasing IGF-1 levels, promoting nerve growth, and producing serum angiotensin and amino acids [135, 136]. Subsequently, this stimulates BDNF receptors, thereby enhancing neuronal connectivity. This neurobiochemical mechanism can influence various regions of the central nervous system, including the forebrain, striatum, hippocampus, cerebral cortex, septal neurons, cerebellum, and motor neurons [139].

In summary, RT combats age-related cognitive decline through mechanisms such as reduced inflammation, enhanced neurogenesis, and increased BDNF as well as IGF-1 levels. It achieves this through various mechanisms, including reducing inflammation, promoting neurogenesis, and increasing the levels of BDNF and IGF-1. These factors work together to enhance cognitive flexibility, improve memory and learning, and potentially slow brain ageing (Fig. 2).

## Exercise and Cognitive Function in Physically Vulnerable Older Adults

Accumulating evidence demonstrates that structured exercise interventions provide significant cognitive benefits for physically vulnerable older adults, particularly those with limited mobility or multiple comorbidities. A systematic review by Sáez de Asteasu et al. [122] demonstrated that exercise interventions in acutely hospitalized older adults improved cognitive function post-discharge. Furthermore, Reparaz-Escudero et al. [3] found that long-term physical exercise combined with multidomain interventions showed promising results in preventing cognitive decline among vulnerable older populations. In hospitalized older adults, multicomponent exercise programs incorporating progressive resistance training and walking exercises have shown significant improvements in cognitive function, particularly in executive function and memory domains [4]. These benefits were observed even with relatively short intervention periods (4–7 days), suggesting that appropriately prescribed exercise can benefit cognitive function even in acute care settings [4].

For frail older adults living in the community, structured exercise programs combining moderate-intensity aerobic and RT have demonstrated effectiveness in improving both physical and cognitive outcomes [100, 101, 122]. However, exercise prescription must be carefully individualized, considering functional capacity, comorbidities, and risk factors [10, 80].

## Implications for Future Research

While the precise exercise dosage in terms of intensity, volume, and type for promoting cognitive health in older adults remains unclear, preliminary evidence highlights a positive relationship between heightened exercise dosage and cognitive health in older adults [14]. Although dose–response investigations are yet to elucidate the exact exercise threshold necessary for cognitive enhancement, it has become apparent that the use of different modes of exercise plays a crucial role [140]. As demonstrated in recent systematic reviews [3, 141], while single-domain exercise interventions generate cognitive benefits, multidomain approaches combining physical exercise with other interventions, such as cognitive training, social engagement, or nutritional strategies may be more effective for maintaining cognitive function in older adults to enhance cognitive resilience and overall brain health. Future research should focus on optimizing these combined interventions and identifying the most effective components. For example, a noteworthy observation from several studies is that a long exercise duration of 6–12 months is required before notable cognitive enhancements become apparent [103, 104]. This progression in cognitive improvement aligns with the advancement of physical function, providing a rationale for advancing exercise dosage through increased intensity, frequency, and duration [14]. While several reviews [44, 79, 142] suggest that a minimum of 6 months of aerobic exercise is necessary to detect cognitive changes, shorter-duration trials have demonstrated positive changes in brain function [65]. Current evidence supports the need for extended duration interventions, including both aerobic and RT, to achieve substantial improvements in cognitive performance [14]. However, it is crucial to recognize that the optimal exercise regimen may vary significantly among older adults, particularly owing to individual needs, preferences, and common comorbidities in this population. The available evidence is insufficient to definitively claim the advantage of one intensity zone over another for cognitive performance [14]. Future research should focus on the complex relationship between exercise intensity and cognitive outcomes, while considering individual circumstances and needs. This approach could inform the design of personalized interventions to enhance the cognitive well-being of the elderly population. Moreover, the focus on aerobic exercise and RT should be expanded to include other promising exercise modalities. For instance, mindfulness-based exercise regimens, which combine mental and physical aspects, have great potential for cognitive improvement [143]. Future studies should explore the cognitive advantages of integrating various types of exercise with cognitive challenges, comparing not only aerobic and RT, but also mindfulness-based and other holistic approaches [19, 25]. When considering concurrent training, research should aim to identify the most effective settings (e.g., group vs. individual, level of supervision) and tailor interventions to individual needs. This could involve comparing different types of concurrent training, varying the intensity and duration of the training, or comparing concurrent training to other forms of exercise with a focus on personalized interventions for older adults [109, 110]. A detailed summary of the impact of different exercise modalities on cognitive health, including training parameters, outcome measures, cognitive domains impacted, mechanisms, and references, has been added in Table 1.

**Table 1:** The impact of different exercise modalities on older adults’ cognitive health

Exercise modality	Training load parameters (frequency [F], intensity [I], time [T], type [T])	Outcome measures/tools used	Cognitive Domains Impacted	Mechanisms of exercise-induced cognitive improvement	References
Low-intensity aerobic exercise	**- F**: 2–5 sessions/week for 6–12 weeks or longer **- I**: 50%–60% MHRR, 3–5 RPE, or 1.6–3.0 METs, pace of 100 steps per minute **- T**: 10–40 min/session **- T**: Continuous and long interval of walking, jogging, cycling	Cognitive Function Assessment Tools: - MMSE - Spatial Memory Tasks (e.g., Stroop Test, Spatial Navigation Tasks) - Mood States (GDS, POMS) - Mental Well-being (Quality-of-Life Questionnaires, e.g., SF-36) - Executive Function (Trail Making Test) Neuroimaging and Biomarkers: - Hippocampal Volume (MRI)	**- Spatial memory**: Enhanced recall of spatial information **- Mood states**: Improved positive mood and reduced depressive symptoms **- Mental well-being**: Increased quality of life and psychological health **- Abstraction and mental flexibility:** Better problem-solving and cognitive adaptability	- Fluctuations in central norepinephrine release - Cortisol release - Provision of neural substrates to the brain - Diminishing oxidative stress and inflammation - Alterations in regional brain volume and activation patterns - Optimization of neural activity - Preservation of specific brain structures - Increased hippocampal volume and neurogenesis - Mitigation of cerebrovascular risk factors - Enhancements in regional cerebral blood flow - Increase BDNF within the hippocampus - Disruption of oxidation–reduction state balance - Reduction in anti-inflammatory factor IL-10	- Ferreira et al. [63] - Rhodes et al. [78] - Varma et al. [58] - Erickson et al. [65] - Muscari et al. [77] - Erickson et al. [21] - Babyak et al. [66]
Moderate-intensity aerobic exercise	**- F**: 3–7 sessions/week for 4 weeks–2 months or longer **- I**: 50%–70% MHRR or VO2max, 3.0–6.0 METs **- T**: 15–45 min/session, totaling 3 h/week **- T**: Brisk walking, water aerobics, dancing, tennis (doubles), biking (< 10 mph), running, cycling, swimming	Cognitive Function Assessment Tools: - Executive Function Tests (WCST, Stroop Test, Trail Making Test Parts A & B) - Verbal Fluency (COWAT, Category Fluency Test) - Episodic Memory (RAVLT, HVLT) - Attention and Processing Speed (DSST, N-back Tests) - Working Memory (Digit Span Forward/Backward, Corsi Block-Tapping Test) Mood and Mental Health: - GDS	**- Executive function:** Improved planning, decision-making, and cognitive flexibility **- Episodic memory:** Enhanced formation and retrieval of new memories **- Depression scores:** Reduced symptoms of depression **- Attention:** Increased focus and sustained attention **- Processing speed:** Faster cognitive processing and reaction times		- Zhidong et al. [76] - Hvid et al. [36] - Guadagni et al. [35] - Sanders et al. [75] - Erickson et al. [65] - Baker et al. [79] - Bixby et al. [74] - Muscari et al. [77]
High intensity aerobic exercise	**- F**: 2–5 sessions/week for 6 weeks to 6 months **- I**: 75–90% MHR, 70–85% MHRR, >6.0 METs **- T**: 20–30 min/session **- T**: HIIT, HIFT	Cognitive Function Assessment Tools: - Working Memory (N-back Tests, Digit Span Tasks) - Executive Function (Tower of London Test, Wisconsin Card Sorting Test) - Processing Speed and Visuospatial Skills (Trail Making Test, Modified Eriksen Flanker Task) - Inhibitory Control (Go/No-Go Tasks) - Reaction Time (Choice Reaction Time Tests) Attention and Verbal Fluency: - Verbal Fluency (Word Generation Tasks)	**- Working memory:** Improved ability to hold and manipulate information **- Executive function:** Enhanced higher-order cognitive control **- Verbal fluency:** Increased word generation and language skills **- Processing speed:** Faster cognitive task performance **- Visuospatial skills:** Improved spatial awareness and navigation **- Inhibitory control:** Better ability to suppress irrelevant responses *greater memory benefits compared to lower-intensity activities		- Xu et al. [25] - Greeley et al. [71] - Simonsson et al. [72] - Dowllah et al. [73] - Hinchman et al. [70] - Penninx et al. [49] - Blumenthal et al. [50] - Durante & Ainsworth [87]
Resistance exercise – Acute effects	- **F**: 2–3 sessions/week for 6 weeks – 6 months or longer - **I**: 40%–80% of 1RM - **T**: 40–60 min/session, 2–3 sets of 8–12 repetitions for 7–10 major muscle groups exercises - **T**: Bodyweight training, weight machines, free weights, elastic bands, medicine balls, plyometrics	Cognitive Function Assessment Tools: - Visuospatial Processing (Mental Rotation Test) - Executive Functions (Stroop Test, Trail Making Test) - Attention and Working Memory (Digit Span Tasks) - Problem-Solving and Cognitive Flexibility (Tower of London Test) - Verbal Fluency (Word Generation Tasks) Memory and Visual Tasks: - Visual Memory Tests - Corsi Block-Tapping Test	**- Visuospatial processing:** Improved spatial reasoning and visualization **- Executive functions:** Enhanced cognitive control and flexibility **- Attention:** Increased alertness and focus **- Working memory:** Better short-term memory retention Problem-solving: Improved analytical thinking and solutions **- Cognitive flexibility**: Greater adaptability in task-switching **- Verbal fluency:** Increased word generation and expression	- Stimulation of VEGF and GH secretion - Down-regulation of peripheral or brain cytokines - Delay of telomere DNA damage and protein aggregation - Inhibition of mitochondrial dysfunction and pro-inflammatory factors - Reduction of lipid peroxidation levels - Regulation of microglial activation - Facilitation of neurogenesis and neural circuitry - Increase in IGF-1 concentration - Increase in stroop test performance and decrease in cortical white matter atrophy - Decrease in the activation index of the prefrontal cortex - Higher levels of BDNF - Release of lactic acid from peripheral areas - Interaction of BDNF with IGF-1	- Washif et al. [93] - Hernández-Gamboa et al. [68] - Lasevicius et al. [91] - Hsieh et al. [95] - Hsieh et al. [94] - Chang et al. [23]
Resistance exercise – Chronic effects		Cognitive Function Assessment Tools: - Executive Function (Stroop Test, Attention Network Test) - Verbal Working Memory (Digit Span Tasks) - Selective Attention and Conflict Resolution (Computerized Tasks) - Response Inhibition (Go/No-Go Tasks) - Information Processing Speed (Digit Symbol Substitution Test) Global Cognitive Function: - MMSE	**- Executive function:** Long-term improvement in cognitive control **- Selective attention:** Enhanced ability to focus on relevant stimuli **- Conflict resolution:** Better management of competing tasks **- Response inhibition:** Improved suppression of impulsive actions **- Information processing speed:** Faster cognitive responses over time **- Attention:** Sustained improvements in focus and concentration **- Working memory:** Long-term retention and manipulation of information		- Chow et al. [98] - Vonk et al. [99] - Tsai et al. [92] - Forti et al. [101] - Kirk-Sanchez et al. [14] - Nagamatsu et al. [103] - Coelho et al. [100] - Liu-Ambrose et al. [104]
Concurrent Strength and Aerobic Exercise	**- F**: 2–3 sessions/week for 8–24 weeks - **I**: - Strength exercises at 50–80% 1RM) - Aerobic exercise at 40–60% VO2max or HRR **T:** 30–60 min/session **T:** Circuit training combining resistance exercises with short rest periods, followed by moderate-intensity aerobic activity such as brisk walking or cycling * There is a controversy in existing research regarding delaying aerobic exercises over resistance exercises or vice versa during the same session, or separating resistance sessions from aerobic sessions during the weekly training program	Cognitive Function Assessment Tools: - Executive Function (Stroop Test, Trail Making Test) - Memory (RAVLT) - Attention (Computerized Cognitive Tests) - Dual-Task Training (Simultaneous Motor-Cognitive Tasks)	**- Executive function:** Combined benefits for cognitive control and flexibility **- Memory:** Enhanced recall and retention of information **- Attention:** Improved focus and dual-task performance **- Dual-task training:** Superior cognitive-motor integration compared to aerobic exercise alone * Dual-task training combined with aerobic exercise might be more effective in boosting cognitive function than just aerobic exercise alone	* Likely a combination of mechanisms from both exercise types - Increased blood flow and delivery of nutrients to the brain - Promotion of neurotrophic factors supporting brain cell growth and plasticity - Improved vascular health, reduced inflammation, and potentially increased hormonal changes influencing brain function	- Castaño et al. [19] - Cheng et al. [67] - Chen et al. [107] - Bossers et al. [108] - Lautenschlager et al. [64]
Common findings		Common impacts across all types of exercise		Common mechanisms across all types of exercise	
		**- Executive function:** Consistent improvement in planning, decision-making, and flexibility **- Attention:** Enhanced focus, alertness, and sustained attention		- Provision of neural substrates to the brain - Mitigation of cerebrovascular risk factors - Inflammation and oxidative stress reduction - Increase in IGF-1 concentration - Increase in BDNF levels	

*FITT* frequency, intensity, time, type, *MHR* maximum heart rate, *MHRR* maximum heart rate reserve, *1RM* one repetition maximum, *HIIT* high-intensity interval training, *HIFT* high-intensity functional training, *BDNF* brain-derived neurotrophic factor, *IGF-1* insulin-like growth factor 1, *VO*_*2max*_ maximal oxygen uptake, *MMSE* Mini-Mental State Examination, *GDS* geriatric depression scale, *POMS* profile of mood states, *WCST* Wisconsin card sorting test, *COWAT* controlled oral word association test, *RAVLT* Rey auditory verbal learning test, *HVLT* Hopkins verbal learning test, *DSST* digit symbol substitution test

## Limitations

Several limitations of this review warrant consideration. First, heterogeneity in exercise intervention protocols across studies made direct comparisons challenging [25, 75, 76]. Studies varied considerably in their definitions of exercise intensities, durations, and frequencies, potentially affecting the interpretation of results [14]. The lack of standardized reporting of exercise parameters in some studies further complicated the synthesis of findings [43, 141]. Second, while we aimed to focus on cognitively healthy older adults, the definition of "cognitive health" varied across studies [16, 25]. Some studies used Mini-Mental State Examination (MMSE) scores as inclusion criteria, while others employed different cognitive screening tools or relied on self-reported absence of cognitive impairment [33, 65]. This variability in cognitive status assessment may have influenced the reported exercise effects. Third, most included studies had relatively short intervention periods (12 weeks to 6 months), with few studies examining long-term effects beyond one year [70, 81]. As demonstrated by recent meta-analyses [3, 75], longer intervention periods may be necessary to fully understand the impact of exercise on cognitive function [14, 101]. Additionally, many studies lacked follow-up assessments after intervention completion, limiting our understanding of the durability of exercise-induced cognitive benefits. Fourth, the diversity of cognitive assessment tools used across studies made it difficult to compare outcomes directly [25, 76]. While some studies focused on specific cognitive domains, others used global cognitive measures, potentially masking domain-specific effects [102, 107]. Furthermore, the sensitivity of different cognitive assessment tools to exercise-induced changes may vary, affecting the detection of intervention effects. Finally, publication bias may have influenced our findings, as studies with positive results are more likely to be published than those showing no effect [25]. Additionally, our review was limited to English-language publications, potentially excluding relevant findings from non-English literature [144].

## Conclusions

**Fig. 4:**
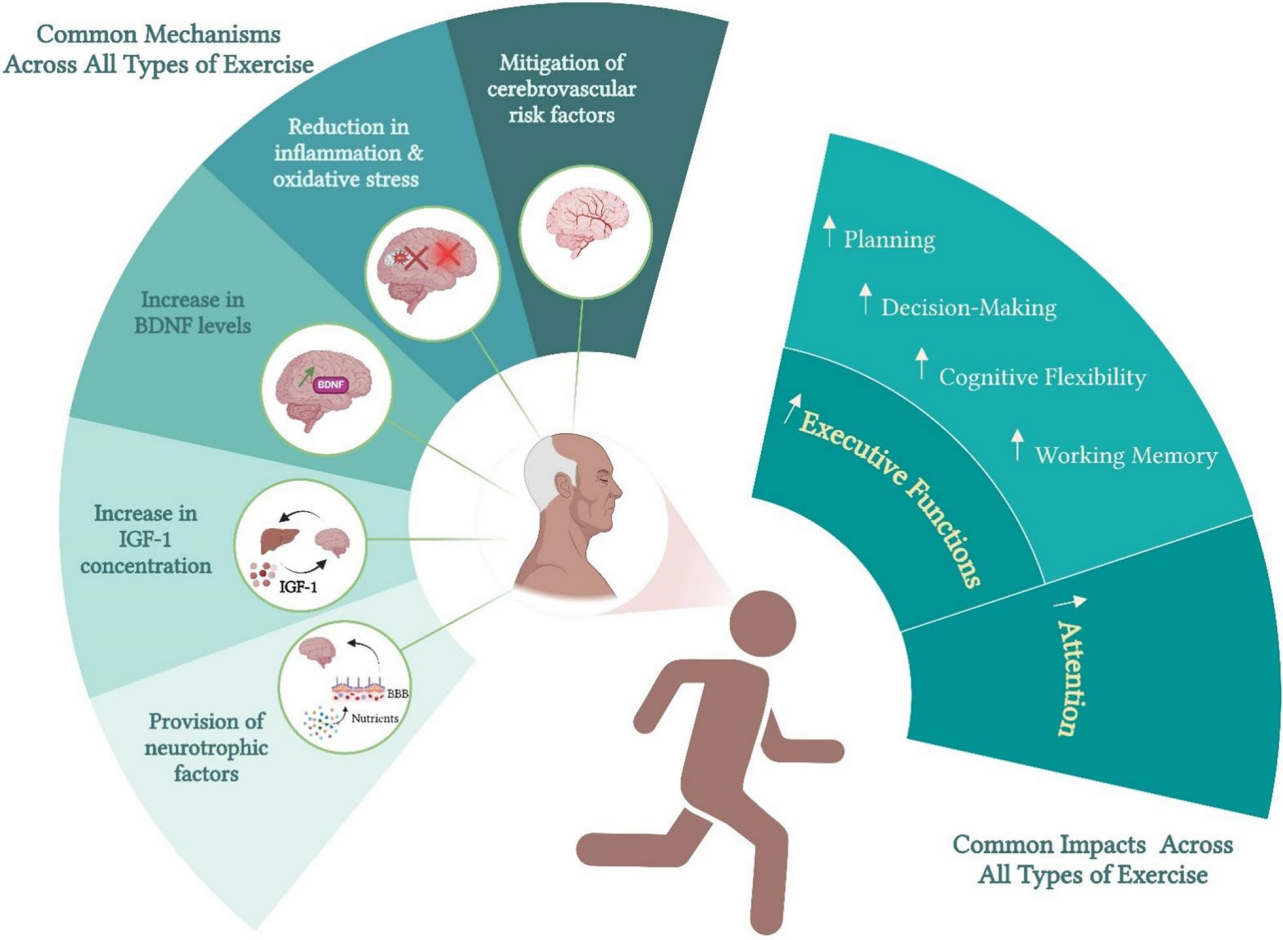
The common impacts of different exercise types on older adults’ cognitive health. *BDNF* brain-derived neurotrophic factor, *IGF-1* insulin-like growth factor 1, *BBB* blood–brain barrier

This review underscores the substantial impact of PA on enhancing cognitive function in older adults, with domain-specific benefits observed across aerobic, resistance, and concurrent training modalities. While the cognitive advantages of moderate-intensity aerobic exercises are well researched, particularly for enhanced executive function, improved memory, and a slower progression of age-related cognitive decline, the efficacy of high-intensity aerobic protocols remains an area of ongoing investigation, with variability in cognitive outcomes potentially influenced by individual fitness levels, metabolic demands, and neurovascular adaptations. These benefits were observed in both older adults with and without mild cognitive impairment. Therefore, designing training programs incorporating moderate-intensity aerobic exercises is highly recommended to improve older adults’ overall cognitive health and well-being. Regarding RT, evidence suggests acute enhancements in cognitive performance, particularly in visuospatial processing and executive functions, with optimal effects observed within a 10 to 20-min time window post-exercise. Chronic RT contributes substantial benefits to neurogenesis and overall brain health. The impact of concurrent training on cognitive function is mixed, with some studies reporting significant cognitive improvements, while others show no changes. However, emerging evidence has highlighted the positive effects of combining concurrent training with cognitive tasks, which often outperform aerobic exercise alone in terms of cognitive benefits. The mechanisms underlying the cognitive benefits of both aerobic exercise and RT include increased cerebral blood flow and oxygen delivery, enhanced neurogenesis, elevated production of BDNF in the hippocampus, and reduced oxidative stress and inflammation (Fig. 4).

## Data Availability

Not applicable.

## Abbreviations

BDNF: Brain-derived neurotrophic factor
DNA: Deoxyribonucleic acid
DTT: Dual-task training
HIFT: High-intensity functional training
HIIT: High-intensity interval training
IGF-1: Insulin-like-growth factor-1
MET: Metabolic equivalent of a task
MHR: Maximum heart rate
MMSE: Mini-mental state examination
PA: Physical activity
RCTs: Randomized controlled trials
RT: Resistance training
RPE: Rate of perceived exertion
VO_2max_: Maximal oxygen uptake
WHO: World Health Organization
1RM: One maximum repetition
